# Comparable Efficacy of a 1-L PEG and Ascorbic Acid Solution Administered with Bisacodyl versus a 2-L PEG and Ascorbic Acid Solution for Colonoscopy Preparation: A Prospective, Randomized and Investigator-Blinded Trial

**DOI:** 10.1371/journal.pone.0162051

**Published:** 2016-09-02

**Authors:** Ji Eun Kwon, Jung Won Lee, Jong Pil Im, Ji Won Kim, Su Hwan Kim, Seong-Joon Koh, Byeong Gwan Kim, Kook Lae Lee, Sang Gyun Kim, Joo Sung Kim, Hyun Chae Jung

**Affiliations:** 1 Department of Internal Medicine, The Armed Forces Capital Hospital, Bundang, Korea; 2 Department of Internal Medicine and Liver Research Institute, Seoul National University College of Medicine, Seoul, Korea; 3 Department of Internal Medicine, Seoul National University Boramae Hospital, Seoul National University College of Medicine, Seoul, Korea; University Hospital Llandough, UNITED KINGDOM

## Abstract

**Background:**

Two liters of polyethylene glycol (PEG) solution administered with ascorbic acid (Asc) can provide efficacy similar to that of a 4-L PEG solution for colonoscopy preparation. In addition, oral bisacodyl (Bis) has been shown to reduce the volume of PEG needed for a bowel preparation with comparable efficacy. This study aimed to compare the efficacy, tolerability and safety of a 2-L PEG solution mixed with Asc versus the combination of Bis, Asc and a 1-L PEG solution.

**Methods:**

This was a prospective, randomized, multi-centre, single-blind, non-inferiority trial. Participants who were scheduled for colonoscopy were included and randomized to receive either 2-L PEG and Asc (2L PEG/Asc group) or 1-L PEG, Asc and 20 mg Bis (1L PEG/Asc + Bis group). The quality of bowel preparation was assessed using the Boston Bowel Preparation Scale. Data regarding tolerance, compliance and adverse events were also gathered.

**Results:**

A total of 187 participants were analyzed; 96 were allocated to the 2L PEG/Asc group and 91 to the 1L PEG/Asc + Bis group. Bowel preparation was adequate in 87.5% (84/96) of patients in the 2L PEG/Asc group and 94.5% of the 1L PEG/Asc + Bis group (86/91, *p* = 0.10). There was no significant difference between the two groups with respect to compliance, tolerability or safety. The patients allocated to the 1L PEG/Asc + Bis group expressed more willingness to repeat the procedure than patients in the 2L PEG/Asc group (*p* = 0.01).

**Conclusions:**

Bowel preparation with Bis and a 1-L PEG/Asc solution is as effective, well-tolerated, and safe as a 2-L PEG/Asc solution.

**Trial Registration:**

ClinicalTrials.gov NCT 01745835; Clinical Research Information Service (CRiS) KCT0000708

## Introduction

Appropriate bowel preparation is critical for the efficacy of colonoscopy.[1,2] If bowel cleansing is inadequate, polyps can be missed, the electrocautery risk increases, scope insertion becomes difficult, the examination takes longer, and the whole procedure may need to be repeated sooner.[3–9] Conventional bowel preparation using a 4-L polyethylene glycol (PEG) solution has been established as standard method and has proven efficacy and safety. However, the relatively large volume and disagreeable taste compared with other lower- volume methods leads to poor compliance and low tolerability. To increase tolerability, satisfaction and compliance, studies have been conducted using a combination of a reduced volume of PEG with an electrolyte lavage solution.[9] The efficacy of 2-L PEG containing ascorbic acid (Asc) was as efficacious as the conventional 4-L PEG method with better compliance.[8,10,11] As a result, the 2-L PEG and Asc combination is now commonly used and shows comparable efficacy with better tolerability compared to 4-L PEG formulations.[9]

Oral bisacodyl (Bis) is an unabsorbable diphenylmethane derivative with bowel-stimulating properties.[12] Bis is capable of producing HAPC (high-amplitude propagated contractions) of the colon, thereby inducing an additional laxative effect.[12,13] The efficacy of bowel cleansing with 15 mg of Bis plus 2 L of PEG was found not to be inferior to the 4-L PEG method and showed better patient compliance and lower rates of adverse events.[14] Moreover, 20 mg of Bis administered with 2-L PEG showed better efficacy, improved patient satisfaction, and shortened bowel preparation time compared to the 4-L PEG method.[7,15] In brief, Bis may not only be an effective method, it could also reduce the volume required for bowel preparation. Meanwhile, recent Korean studies have successfully demonstrated that other reduced-volume preparation methods, such as sodium picosulfate with magnesium citrate (SP/MC), show similar efficacy compared to 2-L of the PEG with Asc.[16] To this end, the present investigators hypothesized that preparation with an even further reduction in volume by Bis addition may demonstrate similar efficacy as the 2-L PEG/Asc solution. Furthermore, the combination of 1-L PEG/Asc + Bis, a relatively low-volume preparation, has not been tested previously.

In this study, the investigators compared the efficacy and tolerability of a 2-L PEG solution containing Asc (PEG/Asc) and the combination of oral Bis 20 mg with 1-L PEG/Asc for colonoscopy preparation. We also included patient preference and adverse events in our analysis of each bowel preparation method.

## Methods

### Study Design and Population

This study was a prospective, randomized, multi-centre, single-blind, non-inferiority trial in adult patients who were scheduled for elective colonoscopy (S1 Checklist). The study was conducted at two centres, Seoul National University Hospital and Seoul National University Boramae Hospital. The study protocol was approved by the institutional review board at each centre (S1 and S2 Files), and written informed consent was obtained from all patients. The trial was registered in an international clinical trials registry, “ClinicalTrials.gov” (NCT 01745835) and one of primary registries in the WHO Registry Network, “Clinical Research Information Service (CRiS), Republic of Korea” (KCT0000708).

The enrolled subjects were adults at least 20 years of age who were scheduled for an outpatient colonoscopy between February and December 2013. Patients were excluded if they had any of the following conditions: ileus, suspected bowel obstruction or toxic megacolon; prior abdominal or pelvic surgery; inflammatory bowel disease; malignancy; severe cardiac disease (heart failure beyond NYHA Class III); chronic obstructive pulmonary disease, interstitial lung disease or uncontrolled asthma; renal failure and on dialysis, decompensated liver cirrhosis; coagulopathy or other hematologic disease with bleeding tendency; dementia or other cognitive disorders; or hypersensitivity to either PEG solution or Bis. Female patients were excluded from the study if they were pregnant or breastfeeding. Patients with long-term use of sedative, anti-spasmodic, prokinetic, laxative or anti-diarrheal medications were also excluded.

### Randomization and Study Protocol

**Fig 1:**
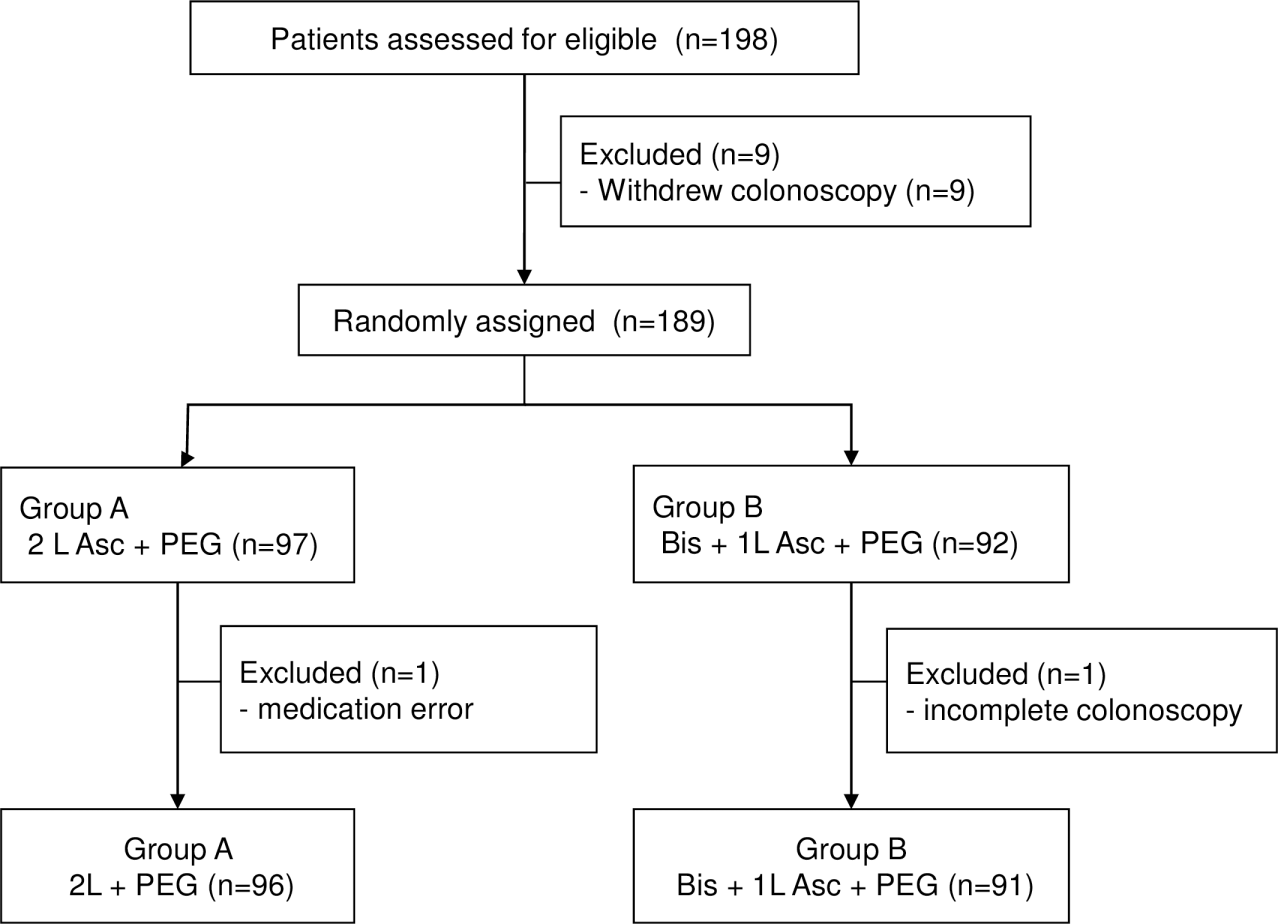
Study flow chart. Asc; ascorbic acid, PEG; polyethylene glycol, Bis; bisacodyl.

The patients were allocated into 2 groups according to a random-number table (Fig 1). Concealment of the randomization was accomplished using independent personnel who were not involved in the colonoscopy procedure or the outpatient department work and were blinded to data collection and analysis. Moreover, the investigators involved in allocation were not allowed to perform any activities associated with data management, including collection and analysis. Patients who met the eligibility requirements were sequentially assigned based on a randomization table. A brochure with dietary regulations was given to each patient instructing them to avoid solid foods, fruits with seeds, and indigestible high-fiber foods for 3 days prior to their colonoscopy, and to eat a soft meal on the day prior to the procedure. In addition, patients were asked to avoid anti-spasmodic drugs, prokinetics, laxatives, dietary fiber and sedatives, all of which could influence gastrointestinal motility.

**Fig 2:**
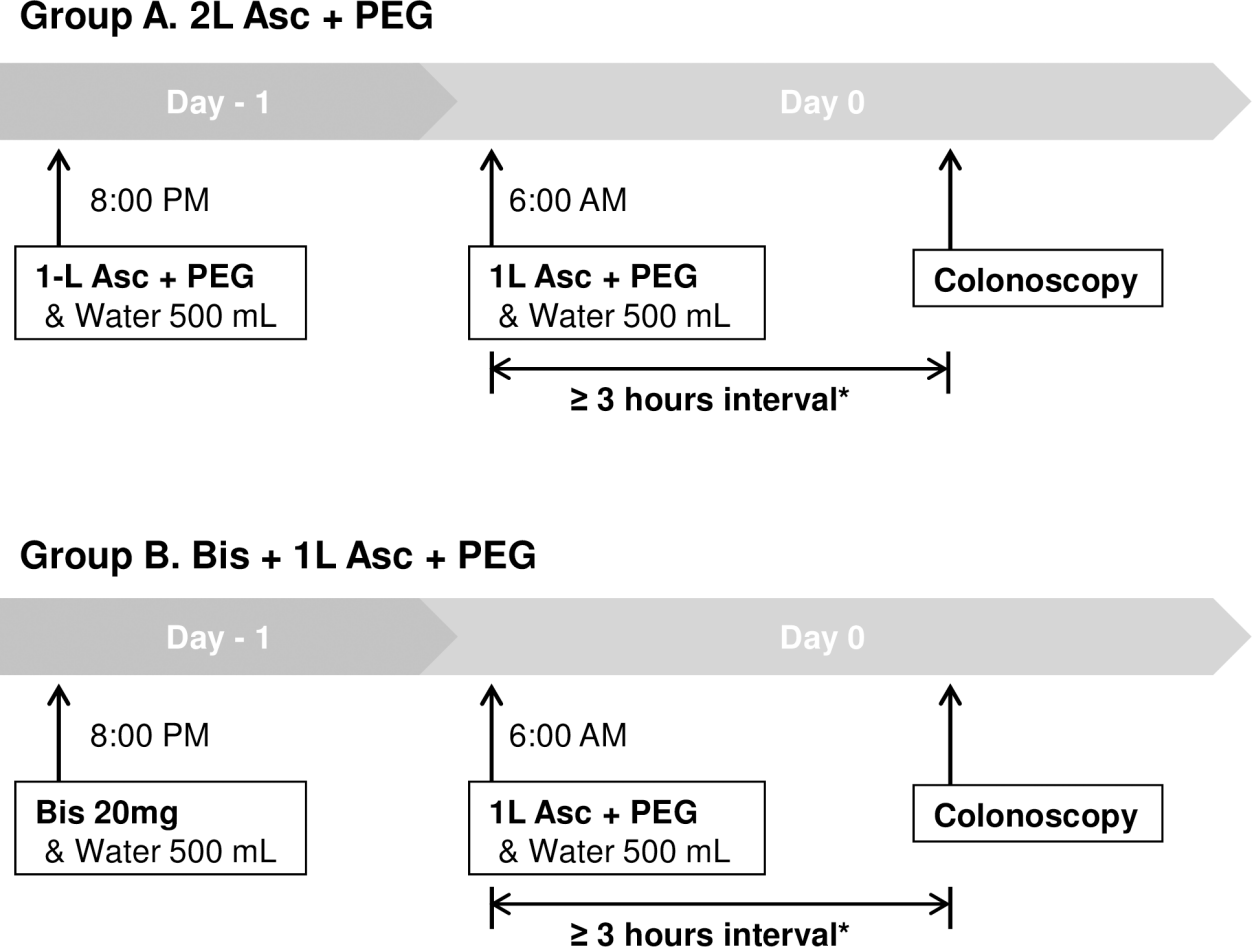
Bowel Preparation Protocols. Asc; ascorbic acid, PEG; polyethylene glycol, Bis; bisacodyl.

Patients who were allocated to the 2-L PEG with Asc (2L PEG/Asc) group took 250 ml of PEG/Asc at 15-min intervals, completing 1-L of the PEG/Asc protocol at 8:00 PM on the day prior to the procedure. The remaining 1-L of the PEG/Asc solution was administered in the same manner at 6:00 AM on the day of the procedure. Patients assigned to the 1-L PEG/Asc with 20 mg Bis (1L PEG/Asc + Bis) group consumed 20 mg of Bis with 500 ml of water at 8:00 PM on the day prior to the colonoscopy and took 1-L of the PEG + Asc solution at 6:00 AM on the day of the colonoscopy in the same way as described above. All of the subjects ingested 500 mL of water for every 1-L of PEG/Asc solution consumed. The patients completed all of the administrations at least 3 hours before the colonoscopy (Fig 2).[17,18]

### Study Endpoints

The primary endpoints were the achievement of preparation adequacy and an overall colon cleansing score of ≥ 6, both assessed by blinded investigators using the Boston Bowel Preparation Scale (BBPS). The present investigators defined an adequate preparation as having BBPS scores of 2 or 3 for all colon segments, whereas if the subject had a BBPS score of 0 or 1 in any colon segment, the preparation was considered inadequate.[19,20] Secondary study endpoints included patient compliance, tolerability, preference and adverse events, which were collected using a questionnaire.

All of the involved endoscopists were unaware of the patient’s bowel preparation method and took pictures that represented bowel preparation state of each segment while the scope was inserted before bowel content removal. The bowel cleansing status was evaluated using the BBPS, a previously validated bowel preparation scoring system based on the summation of the preparation scores from three segments of the colon (right colon, transverse colon and left colon)[15,21]. Two experienced endoscopists, blinded to the preparation method, reviewed all of the colonoscopic images. The two endoscopists were well informed of BBPS scoring system through training programs, from www.cori.org/bbps. Inter-observer variability in the endoscopists' assessment was statistically analysed and the images were cross-checked to confirm the final conclusion. The BBPS is a 9-point scale that ranges from 0 to 9, and the investigators rated the overall preparation status as one of 4 different categories: excellent (BBPS 8–9), good (BBPS 6–7), poor (BBPS 3–5) or inadequate (BBPS 0–2).

On the day of the colonoscopy, participants were asked to complete a questionnaire regarding their prior colonoscopy experience, including their compliance, tolerability, and any adverse events experienced during the bowel cleansing procedure. The subjects also reported the percentage of the preparation they completed (100%, 75~99%, 75% or less). They were asked about the presence of distressing symptoms, such as abdominal pain/discomfort or nausea/vomiting, and severity for each symptoms based on Common Terminology Criteria for Adverse Events, version 4.0 (CTCAE, v4.0). Patients who had a prior colonoscopy were also asked about their preferred bowel preparation method when comparing their prior regimen and the bowel preparation method used in the present study.

### Statistical Analysis

The sample size of this non-inferiority study was based on previously published results.[10,22] The rate of successful bowel preparation was 80% in one Korean study, despite a rate of 88–89% reported in studies of other ethnic groups. Based upon these studies, we assumed that the overall success rate in the 2L PEG/Asc group would be approximately 85%. We hypothesized that a minimum 15% difference in the rates of efficacy between the two preparations would constitute a clinically meaningful difference [23]. Considering a drop-out rate of 10%, the total sample size required was 196 (98 patients in each condition), based on the assumptions of α = 0.025 and β = 0.20. The combination of 1-L PEG/Asc + Bis would be considered non-inferior to 2L PEG/Asc if the one sided 97.5% confidence interval (97.5% CI) for the treatment difference was greater than -15.0%. Treatment difference was calculated by subtracting the percentage of patients achieving an excellent or good preparation status (BBPS score 6 or higher) in the 2L PEG/Asc group from the percentage of those in the 1L PEG/Asc + Bis group.

Continuous variables were expressed as the mean ± SD (range), and categorical variables were presented as absolute values and percentages. All quantitative variables were compared between the two groups using an independent samples t-test. Either the Pearson’s chi-square or Fisher’s exact test was used for categorical variables as appropriate, and a chi-square test was used to investigate trends (ordinal variables). The inter-observer variability was measured by using the interclass correlation coefficent (ICC). All statistical tests were two-sided without adjustment for multiple comparisons. A *p* value less than 0.05 was considered statistically significant. All analyses were performed using the SPSS statistics program (version 19.0; SPSS Inc., Chicago, IL, USA).

## Results

### Baseline Characteristics

The study subjects’ allocation and disposition are described in Fig 1. One hundred and ninety-eight patients provided informed consent. Nine patients withdrew their scheduled colonoscopy before randomization. One patient in the 2L PEG/Asc group was excluded due to a medication error. A patient in the 1L PEG/Asc + Bis group was also excluded due to a failed colonoscopy caused by poor cooperation. A total of 187 subjects completed the study and were included in the final per-protocol analysis.

The baseline characteristics of the study subjects are summarized in Table 1. The age and proportions of male and female participants did not differ significantly. Seventy-eight patients in the 2L PEG/Asc group and 72 in the 1L PEG/Asc + Bis group had undergone a prior colonoscopy, and the bowel cleansing status of the prior colonoscopy was available in 49 patients in the 2L PEG/Asc group and 44 in the 1L PEG/Asc + Bis group. In total, 65.3% (32/49) patients in the 2L PEG/Asc and 72.5% (32/44) patients in 1L PEG/Asc + Bis group had a total colon BBPS score of ≥ 6 during their prior colonoscopy (*p* = 0.44). Four liters of PEG was the most commonly used bowel cleansing protocol in prior colonoscopies.

**Table 1:** Baseline characteristics.

	2L PEG/Asc (n = 96)	1L PEG/Asc+Bis (n = 91)	*p* value
Age±SD, years (range)	56.0±7.7 (23–84)	59.6±12.5 (21–87)	0.09
Male/Female, n/n	51/45	48/43	0.96
Previous colonoscopy, n(%)	78 (81.3%)	72 (79.1%)	0.72
Previous preparation method, n			
4L PEG	47	45	0.68*
NaP	1		
Low-residue diet + 2L PEG	17	12	
Unknown	13	15	
Previous BBPS, n/N (%)†			
Adequate (≥6)	32/49 (65.3%)	32/44 (72.7%)	0.44
Inadequate (<6)	17/49 (34.7%)	12/44 (27.2%)	

SD, standard deviation; PEG, polyethylene glycol; Bis, bisacodyl; NaP, Sodium phosphate; BBPS, Boston Bowel Preparation Scale

*Fisher’s exact test

†2L PEG/Asc (n = 49), 1L PEG/Asc + Bis (n = 44) cases are available for analysis.

### Efficacy of Bowel Preparation

There was no considerable inter-observer variability in the endoscopists' assessment of preparation adequacy (ICC = 0.94, p<0.01) and the images were cross-checked to confirm the final conclusion. The mean total colon BBPS score was 7.6±1.8 in the 2L PEG/Asc group and 7.7±1.7 in the 1L PEG/Asc + Bis group (*p* = 0.73), and bowel preparation scores for each segments were similar in the two groups (Tables 2 and 3). There were no statistically significant differences between the 2L PEG/Asc and 1L PEG/Asc + Bis groups with regard to the preparation rating. The proportion of patients with an adequate bowel preparation (BBPS ≥ 2 for all segments) was not statistically different in the two groups (2L PEG/Asc group: 87.5% vs. 1L PEG/Asc + Bis group: 94.5%, *p* = 0.10). Therefore, the proportion of patients with a BBPS score of 0 or 1 in any colon segment, i.e. candidates for early repeat colonoscopy, did not differ between groups. The one sided 97.5% CI for treatment difference in overall colon cleansing between the bowel preparations was greater than—15.0%; therefore, the efficacy of 1L PEG/Asc + Bis was determined to be non-inferior to 2L PEG/Asc in overall cleansing of the colon (Table 3). Also, 1L PEG/Asc + Bis was non-inferior to 2L PEG/Asc in cleansing the right, transverse, and left colon as measured by the BBPS.

**Table 2:** Boston Bowel Preparation Scale scores and preparation rating.

	2L PEG/Asc (n = 96)	1L PEG/Asc+Bis (n = 91)	*p* value
**BBPS, whole colon, mean±SD**	7.6±1.8	7.7±1.7	0.73
BBPS, right colon, mean±SD	2.4±0.7	2.5±0.6	0.26
BBPS, transverse colon, mean±SD	2.6±0.6	2.6±0.5	0.67
BBPS, left colon, mean±SD	2.7±0.5	2.6±0.5	0.51
**BBPS, preparation rating, n(%)**			
Excellent (BBPS 8–9)	61 (63.6%)	56 (61.5%)	0.07*
Good (BBPS 6–7)	23 (24.0%)	31 (34.1%)	
Poor (BBPS 3–5)	11 (11.5%)	3 (3.3%)	
Inadequate (BBPS 0–2)	1 (1.0%)	1 (1.1%)	

*Fisher’s exact test

BBPS, Boston Bowel Preparation Scale.

**Table 3:** The Efficacy of Bowel Preparation.

	2L PEG/Asc (n = 96)	1L PEG/Asc+Bis (n = 91)	treatment difference	One-sided 97.5% CI
**BBPS, total ≥ 6 (above good), n(%)**	84 (87.5%)	87 (95.6%)	8.1	0.3
**Achievement of adequacy, n(%) (BBPS ≥2, for all segments)**†	84 (87.5%)	86 (94.5%)	7.1	-1.1
BBPS≥2, right colon, n	86	87	6.0	-1.4
BBPS≥2, transverse colon, n	93	89	0.9	-3.7
BBPS≥2, left colon, n	94	89	-0.1	-4.3

BBPS, Boston Bowel Preparation Scale

†Patients with BBOS scores of 2 or 3 for all colon segments.

### Tolerability and Compliance

Patient tolerability and compliance with the bowel preparations are described in Table 4. More than 90% of patients in both groups successfully performed the preparation protocol according to the pre-set schedule. Moreover, some patients in both groups achieved completion rates above 95%. More than 80% of the patients in both groups complied with the dietary recommendations, including avoidance of solid and high fiber foods for 3 days prior to the procedure. The patients’ willingness to repeat the study bowel preparation and preference for this method were assessed in the 78 patients in the 2L PEG/Asc group and 72 patients in the 1L PEG/Asc + Bis group who had prior experience with a colonoscopy (Table 4, lower section). Among these patients, those belonging to the 1L PEG/Asc + Bis group were overwhelmingly more willing to repeat the study preparation (61/72, 84.7%), compared to 66.7% (52/78) of the 2L PEG/Asc group subjects (2 L Asc + PEG) (*p* = 0.01). Additionally, significantly more patients who used the 1L PEG/Asc + Bis method stated that they preferred the current over their prior method because it was “easier,” as compared to that of the 2L PEG/Asc group (*p* < 0.01).

**Table 4:** Tolerability and Safety.

	**2L PEG/Asc (n = 96)**	**1L PEG/Asc+Bis (n = 91)**	***p* value**
Completion rate, n (%)*	94 (97.9)	90 (98.9)	1.00
On time administration, n (%)	91 (94.8)	88 (96.7)	0.72
Adequate Dietary regulation, n (%)†	78 (81.3)	76 (83.5)	0.68
Presence of Adverse event, n (%)	30 (31.3%)	32 (35.2%)	0.57
*Nausea*	9 (9.4%)	17 (18.7%)	0.07
*Vomitting*	6 (6.3%)	1 (1.1%)	0.12
*Abdominal pain*	3 (3.1%)	6 (6.6%)	0.32
*Bloating*	10 (10.4%)	12 (13.2%)	0.56
*Dizzness*	4 (4.2%)	5 (5.5%)	0.74
*Anal irritation*	0 (0.0%)	1 (1.1%)	0.49
	**2L PEG/Asc (n = 78)** ^#^	**1L PEG/Asc+Bis (n = 72)** ^#^	***p* value**
Willingness to repeat	52 (66.7)	61 (84.7)	0.01
Reason to prefer the present method, n (%)‡			
*Low volume*	46 (59.0)	53 (73.6)	0.06
*Easier*	12 (15.4)	29 (40.3)	<0.01
*Better taste or smell*	20 (25.6)	25 (34.7)	0.23
*Fever adverse event*	5 (6.4)	10 (13.9)	0.13

* Solution intake exceeded 75%

# Patients with prior colonoscopy experience

†Avoidance of solid/high fiber food for 3 days

‡ Questionnaire included multiple choice answers.

### Adverse Events

A total of 62 subjects (30 in the 2L PEG/Asc group and 32 in the 1L PEG/Asc + Bis group) experienced adverse events, and the difference in these rates did not reach statistical significance (*p* = 0.57). Nausea and bloating were the most common adverse events in both groups. None of the individual side effects observed in either group showed a statistically significant difference in their occurrence rate. There were no grade≥3 gastrointestinal disorders, according to CTCAE, v4.0. Therefore, we observed no severe adverse events that required hospital admission.

## Discussion

We found that there was no difference in the adequacy of bowel preparation with use of a 1L PEG/Asc + Bis or a 2L PEG/Asc preparation. Furthermore, the achievement of adequacy using the 1 L PEG /Asc + 20 mg Bis method appeared to be superior to the 2L PEG/Asc (94.5% vs. 87.5%, respectively). Early repeat colonoscopies also seemed to be required in fewer patients in the 1L PEG/Asc + Bis group (12.6% vs. 5.4%). Although these observations did not reach statistical significance (*p* = 0.10), the present authors suggest that 1L PEG/Asc + Bis may be another effective method for preventing early repeat colonoscopies. In particular, for patients distressed by the high volume of preparation agent used in traditional methods, this novel oral 20 mg Bis and 1 L PEG with Asc agent could be a good alternative or salvage preparation method. Notably, patient tolerance to the 1L PEG/Asc + Bis was not inferior to that of the 2L PEG/Asc solution. Most subjects in the present study also took the preparation agents on time, and both groups demonstrated very high completion rates for their respective preparation agents, above 95%. It may be inferred that both of the low-volume methods presented in this study show the tolerability necessary to achieve adequate preparation results. The patients who used the 1L PEG/Asc + Bis solution were significantly more willing to repeat the procedure if necessary. This implies that a low-volume preparation method based on a 1-L PEG solution could improve satisfaction compared to the 2-L PEG method. More patients expressed a preference for the low-volume method due to the ease of use, better taste or smell and fewer adverse events.

The ideal bowel preparation method should provide an opportunity to visualize the entire colonic mucosa while being acceptable to and safe for patients. The PEG-based bowel preparation solution is regarded as the gold standard for colonoscopy preparation.[24] However, a significant proportion of patients are unwilling to repeat the large-volume PEG regimen.[22,25,26] Researchers have investigated several different combinations of low-volume PEG and medications, such as sodium phosphate, senna, magnesium, Bis, and Asc, to enhance the bowel cleansing effect. However, these studies have often shown conflicting or inconclusive results.[8,10,11] [25–27] The combination of high-dose Asc with 2 L of PEG is recognized as an effective and safe bowel preparation method, is comparable to the conventional 4 L PEG method, and 2L PEG/Asc formulations are currently approved by the Food and Drug Administration (FDA) of the United States and many other countries.[9,27] However, a considerable number of patients found that even 2 L was too great a volume to take. Therefore, the present investigators designed a study with the aim of reducing the required PEG volume to less than 2 L via the addition of oral Bis. A study conducted in Australia showed that 15 mg oral Bis reduced the required volume of PEG from 4 to 2 L with improved patient acceptability.[8] In addition, 20 mg oral Bis was shown to improve the bowel cleansing effect as well as patient satisfaction, and the 2-L PEG plus 20 mg Bis regimen was as efficacious as the 4-L PEG regimen.[11,28] In contrast, 10 mg Bis did not influence the adequacy of bowel cleansing, and 10 mg Bis administered with a 2-L PEG solution was not as effective as a 2-L PEG/Asc solution.[10,29] A recent study performed in South Korea also demonstrated that the quality of bowel preparation in hospitalized patients was unchanged by the addition of 10 mg oral Bis.[24] In the studies described above, Bis administration did not significantly increase the frequency or severity of adverse events. In addition, studies using Asc with PEG and/or oral Bis showed no cause for concern in the biochemical and hematologic parameters analyzed.[22,30] Based on these previous results, the present investigators determined 20 mg to be the optimal dosage of oral Bis. Under this assumption, the present investigators hypothesized that the addition of 20 mg Bis might bring sufficient efficacy to half the usual dosage of PEG/Asc. This was the first study to demonstrate the efficacy of 1-L PEG/Asc + Bisacodyl compared with 2L PEG/Asc.

In the present study, there was no intergroup difference in the proportion of adverse drug events. In the 1L PEG/Asc + Bis group, none of the patients reported vomiting, although more patients experienced nausea and abdominal pain and/or discomfort in comparison to 2L PEG/Asc group, without a statistically significant difference between groups. Oral Bis may have cause these symptoms by stimulating the nerve endings in the walls of the large intestine to promote intraluminal secretion of water and electrolytes and enhance visceral muscles contraction.[31] Several cases of possible bisacodyl-induced ischemic colitis have been reported; however, the pathogenesis has not be clarified.[32,33] Nonetheless, none of our study participants experienced serious adverse events, including colonic ischemia.

There are several limitations of the present study. The study population consumed an additional liter of water with the bowel preparation medicines; therefore, the total volume of fluid administration was 3 L in the 2L PEG/Asc group and 2 L in the 1L PEG/Asc + Bis group. In addition, the investigators could not suggest the relationship between mean procedure time and the quality of bowel preparation. The institutions in this study were tertiary referral hospital in South Korea, and total procedure times were enlongated mainly due to additional procedures such as polypectomy. Unfortunately, our study could not provide more detailed information regarding other variables that could affect the quality of a bowel preparation, such as diabetes, narcotic use, indications for the procedure, BMI and constipation. The lack of generalizability to other races and co-morbidities are other limitations of this study. Additional studies should follow.

In conclusion, the present study suggests that 20 mg oral Bis can reduce the volume of the PEG/Asc solution from 2 L to 1 L in colonoscopy preparations with comparable efficacy and no significant adverse events. Therefore, 1L PEG/Asc + Bis can be considered a reasonable alternative to the traditional 4 L PEG or 2 L PEG/Asc methods for bowel preparation.

## Supporting Information

S1 Checklist CONSORT 2010 checklist of information to include when reporting a randomised trial. (DOC)

S1 File Study protocols (Korean version). (DOCX)

S2 File Study protocols (English version). (DOCX)
