# Supplementary material for: Comparable Efficacy of a 1-L PEG and Ascorbic Acid Solution Administered with Bisacodyl versus a 2-L PEG and Ascorbic Acid Solution for Colonoscopy Preparation: A Prospective, Randomized and Investigator-Blinded Trial
Source: PLoS One. 2016 Sep 2;11(9):e0162051. doi: 10.1371/journal.pone.0162051 (PMC5010253; doi:10.1371/journal.pone.0162051)
Supplement: S1 File — (DOCX) [file pone.0162051.s002.docx]

# **연구계획서**

**대장 내시경 전처치로 2L Coolprep® 하제 단독복용과 1L Coolprep® 하제 및 bisacodyl 복합요법의 장정결도 비교 연구**

**Comparison between 2L Coolprep® and Combination of 1L Coolprep® and bisacodyl as bowel preparation for colonoscopy**

**일시: 2012년 7월**

**서울대학교 병원 소화기 내과**

**교수 임 종 필**

# **연구계획서 개요**

| 연 구 제 목 | 대장내시경 전처치로, 2L CoolprepⓇ (PEG+Ascorbic acid) 하제 단독복용과 1L CoolprepⓇ 하제 및 bisacodyl 복합요법의 장정결도 비교 연구 |
| --- | --- |
| 연 구 목 적 | 대장내시경 전 전처치는 대장내시경의 효율을 높이는 데 중요한 것으로 알려져 있다. 또한 전처치용 하제로, 2L PEG+ascorbic acid 가 4L PEG 비교하였을 때 정결도가 뒤지지 않음이 잘 알려져 있다.  본 연구에서는 대장내시경 전처치용 하제로 새로나온 2L CoolprepⓇ 재제를 이용하여, 2L CoolprepⓇ하제 단독요법과 1L CoolprepⓇ 하제 및 경구 bisacodyl 복합요법의 효과를 비교하고자 한다. |
| 연 구 기 관 | 서울대학교병원 소화기내과  서울특별시립보라매병원 소화기내과 |
| 연구책임자 | 내과 김지원 교수  내과 임종필 교수 |
| 연 구 대 상 | IRB 승인시점 이후 서울대학교병원 소화기내과 및 서울특별시립보라매병원 소화기내과 외래에서 대장내시경을 처방 받는 환자 |
| 연 구 기 간 | IRB 승인일 이후 ~ 2013년 7월 31일 |
| 연 구 방 법 | 연구 기간 동안 서울대병원 및 서울특별시립보라매병원 소화기내과 외래에서 대장내시경을 처방 받아 시행 예정인 환자를 대상으로, 무작위 배정하에, 2L CoolprepⓇ 하제만을 복용한 환자를 대조군으로, 1L CoolprepⓇ 하제 및 경구 bisacodyl의 복합요법을 받은 환자를 실험군으로 한다. 시술자는 각 환자의 처방을 모르는 상태에서, 대장내시경시 장정결의 정도를 평가하도록 하고, 각 군에서의 장정결정도, 삽입 및 회수에 걸린 시간, 부적절한 장정결로 재검이 필요한 예의 수, 맹장 도달율, 선종 발견율을 비교 분석하고자 한다. 또한 설문조사를 통해 환자의 장정결 처치에의 순응도 및 선호도를 비교해보고자 한다. |
| 기대효과 및  예상결과 | 이미 4L PEG 하제 단독 복용법 보다 2L 하제 및 bisacodyl 복합사용법이 환자 순응도를 상승시키면서 그 효과는 뒤지지 않는다는 것이 잘 알려져 있다. 본 연구에서는 하제 복용의 용량을 더 줄임으로써 환자의 순응도를 증가시킬 수 있고, 장정결도의 차이는 없다는 것을 보고자 한다. 이를 통해, 하제 복용법에 대한 환자 순응도 증가를 유도하여, 그로 인한 대장내시경 검사결과의 질적 향상을 도모할 수 있을 것으로 기대한다. |

**목 차**

1. 연구 제목 -------------------------------------------------------------------------- 4

2. 실시 기관명 및 주소 --------------------------------------------------------------- 4

3. 책임연구자 및 공동연구자 -------------------------------------------------------- 4

4. 연구 배경 및 목적 ----------------------------------------------------------------- 5

5. 예상 연구 기간 -------------------------------------------------------------------- 6

6. 연구 방법

6.1 연구 방법 개요 ----------------------------------------------------------- 6

6.2 시험군과 대조군의 복약 방법 -------------------------------------------- 7

6.3 피험자의 선정 기준 및 제외 기준 -------------------------------------- 8

6.4 목표 피험자의 수 및 산출 근거 -------------------------------------- 8

6.5 무작위 배정----------------------------------------------------------------- 11

6.6 통계 분석 원칙 및 방법 ------------------------------------------------- 12

7. 관찰 항목 및 관찰 검사 방법

7.1 연구 진행 일정표 ------------------------------------------------------ 13

7.2 관찰항목 ------------------------------------------------------ 14

8. 평가기준, 평가 방법 및 해석 방법 ------------------------------------------------- 14

9. 연구의 윤리성 확보를 위한 방안 -------------------------------------------------- 16

10. 참고 문헌 ------------------------------------------------------------------------ 17

**1. 연구제목**

**국문 제목**: 대장 내시경 시행전처치로 2L CoolprepⓇ 하제 단독복용과 1L CoolprepⓇ 하제 및 bisacodyl 복합요법의 장정결도 비교 연구

**영문 제목**: Comparison between 2L CoolprepⓇ and combination of 1L CoolprepⓇ and bisacodyl as bowel preparation for colonoscopy

**2. 실시 기관 명 및 주소**

기관 명칭 : 서울특별시립보라매병원

주 소 : 서울특별시 동작구 보라매로 5길 20

기관 명칭 : 서울대학교병원

주 소 : 서울특별시 종로구 대학로 101

**3. 책임연구자 및 공동연구자**

책임연구자 : 서울특별시립보라매병원 내과 부교수 김 지 원

    연락처 : 서울특별시 동작구 보라매로 5길 20 (02) 870-2251

책임연구자 : 서울대학교병원 내과 임상조교수 임 종 필

    연락처 : 서울대학교 의과대학 간연구소 (02) 740-8112

공동연구자 : 서울특별시립보라매병원 내과 진료조교수 고 성 준

    연락처 : 서울특별시 동작구 보라매로 5길 20  (02) 870-2254

공동연구자 : 서울대학교병원 내과 임상강사 권 지 은

연락처 : 서울대학교 의과대학 간연구소 (02) 740-8112

공동연구자 : 서울특별시립보라매병원 내과 임상강사 김 수 환

연락처 : 서울특별시 동작구 보라매로 5길 20  (02) 870-2254

**4. 연구 배경 및 목적**

대장내시경시 적절한 장 정결은 대장 점막을 완전히 관찰하는 데 필수적이다[1-2]. 장 정결이 부적절할 경우 병변을 놓치는 원인이 될 수 있으며, 시술이 어렵고 오래 걸리게 하며, 합병증의 위험도를 증가시키고, 이른 시기에 검사를 반복해야 하는 문제를 유발할 수 있다.

통상적으로 외래에서 처방되는 4L PEG (Polyethylen glycol, ColyteⓇ) 용액 복용법은 냄새와 맛이 역하고 복용량이 많아 환자의 순응도를 떨어뜨리게 된다. 대장내시경전 적절한 장 정결을 위하여 PEG 용액은 2L 로 줄이는 대신 경구 약제를 함께 복용하는 방법에 대해 많은 연구가 이루어져 왔다. 특히, 2L PEG 용액에 Ascorbic acid 또는 bisacodyl 을 더하여 복용하는 경우, 그 효과가 4L PEG 용액의 단독 복용법에 뒤지지 않으면서 환자의 순응도를 개선시킬 수 있음이 여러 차례 보고된 바 있다[3-7]. 이에 최근 국내에 출시된 2L PEG 및 Ascorbic acid 복합재제인 CoolprepⓇ이 기존에 통상적으로 사용되었던 4L PEG 용액, ColyteⓇ 에 뒤지지 않을 것으로 기대된다[5].

자극성 하제인 bisacodyl은 흡수가 되지 않는 diphenylmethane으로, 기존 연구에서 bisacodyl 10mg을 PEG와 병용하여 투여하였을 때, PEG 단독 투여와 비교하여 장 정결의 상태를 효과적으로 개선시키지는 못하였다[8]. 그러나 bisacodyl 15mg과 2L PEG 용액 병용 투여를 4L PEG 용액 단독 투여와 비교하였을 때, 장 정결의 상태에는 유의한 차이가 없었으나 bisacodyl을 병용 투여한 환자의 순응도가 더욱 우수하였으며 오심 등의 부작용이 적었다[3]. 또한 bisacodyl 20mg을 2L PEG 용액과 병용하였을 때, PEG 4L를 단독으로 투여한 것에 비하여 환자의 만족도를 높이고 전처치의 시간을 줄일 뿐만 아니라, 장 정결 상태도 더욱 개선시킨다는 보고도 있었다[4].

이러한 연구결과를 바탕으로, 최근 국내에 출시된 PEG 및 Ascorbic acid 복합재제인 CoolprepⓇ 에 Bisacodyl을 추가하여 복용하면, 2L Coolprep 단독 복용시보다도 하제 복용량을 줄이면서 장 정결 효과는 비슷하거나 뒤지지 않을 것으로 기대할 수 있다. 이에, 저자들은 2L CoolprepⓇ 단독 복용군과, 해당 제재 1L 와 경구 bisacodyl의 복합복용군의 장정결의 효과를 비교해보고자 한다.

**5. 예상 연구 기간**

IRB 승인 시점 이후 ~ 2013년 7월 31일

**6. 연구 방법**

**6. 1. 연구 방법 개요**

본 연구는 다중기관, 전향적 무작위 배정 연구로 고안되었다. 서울특별시립보라매병원 소화기내과 및 서울대학교병원 소화기내과 외래 외래에서 대장내시경을 처방 받는 만20세 이상의 환자들을 대상으로 자의에 의해 피험자가 연구에 참여할 것을 서면으로 동의하면, 선정 기준에 적합하고 제외 기준에 해당하지 않는 피험자에 한하여 대장내시경 처방시 2L CoolprepⓇ만을 복용하는 군과 1L CoolprepⓇ 복용 및 경구 bisacodyl 5mg 4정 (20mg)을 복용하는 군으로 1:1 의 비율로 무작위 배정을 한다.

**6. 2. 시험군과 대조군의 복약 방법**

사용되는 하제는 PEG 3350, 무수황산나트륨, 염화나트륨, 염화칼륨, 아스코르브산, 아스코르브산 나트륨 복합재제인 2L CoolprepⓇ (TaeJoon Pharmaceuticals, Seoul, Korea)으로 한다. 관련된 불편 혹은 위험성으로는, 피로감, 복부 팽만감이나 복부 통증, 항문 자극, 구역, 구토, 어지러움, 두통 등이 있다. 1L 기준으로 성분∙함량은 다음과 같다.

1L 기준함량 : 염화나트륨 2.691g, 염화칼륨 1.015g, 무수황산나트륨 7.5g, 폴리에틸렌글리콜3350 100.0g, 아스코르브산 4.7g, 아스코르브산나트륨 5.9g

대장운동 촉진제는 Dulcolax-SⓇ (Boehringer Ingelheim ,Seoul, Korea)을 사용한다. 관련된 불편 혹은 위험성으로는, 1% 미만에서 전해질 및 수분 불균형을 초래할 수 있고, 그 외 경도의 복부 통증이나 불편감, 구역, 구토, 직장의 화끈거리는 느낌, 어지러움증이 있다. 1정 기준으로 성분∙함량은 다음과 같다.

1정 기준함량 : Bisacodyl 5mg, docusate sodium 16.75mg

두 약물간의 상호작용은 알려져 있지 않으며, 이는 Launch Lexi-Interact™ Drug Interactions Program을 이용하여 확인하였다.

대장 검사 전날 저녁식사는 미음으로 제한한다.

□ 대조군의 복용방법

검사 전날 저녁에 미음으로 식사를 하고, 저녁 8시부터 1 L CoolprepⓇ 을 1시간에 걸쳐 복용한다. 즉, 15분마다 CoolprepⓇ 용액을250ml 한 컵씩 총 1시간에 걸쳐 복용하고, 모두 복용 후 추가로 물을 500ml 복용한다. 검사 당일, 검사 3시간 전에 마칠 수 있도록 1시간 동안 1L CoolprepⓇ을 추가 복용한다. 검사 전날과 마찬가지로 15분마다 250ml 한 컵씩 복용하고, 모두 복용 후 추가로 물을 500ml 복용한다. 이 모든 과정은 검사 2시간 전에 마칠 수 있도록 한다.

□ 실험군의 복용방법

검사 전날 저녁 미음으로 식사를 하고, 식후 2시간에 대장운동 촉진제인 Bisacodyl (Dulcolax-SⓇ) 4알(20 mg)을 복용한다. 검사 당일에 1L의 CoolprepⓇ 용액을 1시간에 걸쳐 추가 복용하되, 검사 3시간 전에 마치도록 한다. 1L의 CoolprepⓇ 용액 복용법은 대조군과 마찬가지로 15분마다 CoolprepⓇ 용액을250ml 한 컵씩 총 1시간에 걸쳐 복용한 후 추가로 물을 500ml 복용하도록 한다. 이 모든 과정은 검사 2시간 전에 마칠 수 있도록 한다.

**6. 3. 피험자의 선정기준 및 제외기준**

□ 선정 기준

(1) 본 연구 참여에 동의하고, 동의서에 자의로 서명한 자

(2) 만 20세 이상 성인 남녀

(3) 연구 시작 시점부터 서울대학교 병원 및 시립보라매병원 소화기내과 외래에서 대장내시경을 처방 받는 환자

□ 제외 기준

(1) 위, 소장 혹은 대장의 외과적 절제 수술(다만, 충수 절제술은 제외)의 과거력이 있는 환자

(2) 염증성 장질환(크론병 또는 궤양성 대장염)의 과거력 혹은 현재 병이 있는 환자

또는 임상적으로 강력히 의심되는 환자

(3) 위장관의 기능적 혹은 해부학적 폐색증이 있는 환자 (위무력증, 위날문폐색증, 가성 장폐색, 독성거대결장증, 등)

(4) 악성 종양의 과거력이나 현재 병이 있는 환자

(5) 중대한 심혈관계 질환, 호흡기계 질환, 신장질환, 간질환, 혈액계 질환을 합병하고 있는 환자

(6) 치매나 인지 장애, 의식 장애가 있는 환자

(7) 본시험의 약효 평가에 영향을 미친다고 생각되는 약제 (진경제, 소화관운동개선제, 지사제, 완화제, 신경안정제)를 지속적으로 복용하는 환자

(8) 사용되는 약제에 과민반응을 보이는 환자

(9) 임산부 및 수유부

(10) 본 연구에 동의하지 않는 환자

(11) 그 외에, 연구책임자 또는 연구시행자가 본 연구의 대상으로 부적합하다고 판단한 환자

□ 시험중지 및 탈락 기준, 분석 제외 기준

(1) 선정기준/제외기준에 위배된 경우

(2) 피험자에게 중대한 이상 반응(Serious Adverse Events)이 발생한 경우 혹은 이상반응(Adverse Events)으로 인해 피험자가 시험 중단을 요구하는 경우

(3) 피험자가 연구 참가 동의를 철회한 경우

(4) 피험자의 추적이 안 되는 경우

(5) 피험자가 전처치 하제나 경구 약제 복용에 문제가 있는 경우

(6) 임상시험 기간 동안 임상시험 연구자의 지시 없이 연구 결과 판정에 영향을 미칠 수 있는 약물 등을 복용한 경우

(7) 기타 시험자의 판단에 의해 연구의 진행이 적합하지 못하다고 판단되는 경우

**6. 4. 목표 피험자의 수 및 산출 근거**

본 연구의 목적은 CoolprepⓇ 1L + Bisacodyl 군에서의 장정결도가 CoolprepⓇ 2L 군에 비해 뒤지지 않음을 보이고자 하는, 비열등성 임상시험이다 (단측 검정). 각 군당 할당비 1:1이며, 동등성은 15% 이내로 설정하였다.

4L PEG 하제 단독 복용과 2L PEG 및 Ascorbic acid 복합복용을 비교한 연구에서는 장정결도의 성공률이 94.8%와 88.9%[5], 2L PEG 및 Ascorbic acid 복합복용과 2L PEG 및 Bisacodyl 복합복용을 비교한 연구에서 장정결도의 성공률이 각각 92%, 89%에 달하는 것으로 보고되었다[7] . 국내의 연구에서는 2L PEG와 bisacodyl 20mg를 병용 투여하는 군에서는 79.9%, 4L PEG를 단독 투여하는 군에서는 96.6%로 장정결도 차이는 있었으나, 시술자가 용종 절제술을 시행하는 데 있어서 영향을 주지는 않았으며, 환자의 만족도가 좋고 복통을 제외한 부작용의 빈도가 적었다고 보고되기도 했다[6]

Coolprep 의 양과 성분이, 2L PEG 및 Ascorbic acid 복용시와 차이가 없으므로, 이전 문헌에서 2L PEG + Ascorbic acid 군의 장정결 성공률을 참고하였고, 장정결 성공률이 외국의 문헌에서는 88%, 89%에 달하나, 국내 보고에서는 80% 밖에 되지 않음을 고려하여 P_t_ = 85%로 선정하였고, 동등성을 15% 이내로 선정하였다. 또한, Alpha level 0.025, 검정력(power) 0.8 로 선정하여 실험군이 대조군에 비해 열등하지 않다는 것을 검증하기 위한 환자수를 다음 수식을 통해 계산하였다. (P_t_= 0.85, ε =0.15, α=0.025, β=0.8)

P_t_ : 시험군에서의 성공률, P_c_ : 비교균에서의 성공률, 두 집단의 할당비가 λ (시험군:대조군=1: λ)이고 비율 P_t_ P_c_ 를 예상하고, 최대 허용 차이가 ε가 주어지는 경우 시험군의 표본수는 다음의 형태로 주어진다.


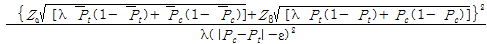


만약 P_t_ =P_c_ =P 라고 가정하면 위의 식은 다음과 같이 간략하게 쓸 수 있다.


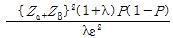


식에 대입해 보면, 다음과 같다.
$\frac{(1.96 + 0.84)^{2} x (1+1) x 0.85 x (1-0.85)}{1 x ( 0.15 )^{2}}$ ≒ 88.853

계산 결과 각 군당 약 89명의 환자가 필요하다. 탈락율을 10%로 계산하여 각 군당 98 명씩, 총 196명의 환자를 enroll 하기로 계획하였다.

**6. 5. 무작위 배정**

본 연구에 필요한 피험자 수는 탈락율 (10%)을 고려하여 각 군당 98명씩 총 196명이다. 무작위배정표는 의학연구협력센터 (Medical Research collaborating center, MRCC)에 의뢰하여 제작하고 관리약사에게 직접 전달되도록 한다. 관리약사는 대조군 및 치료군을 순차적으로 배정하여 대장내시경 전처치제를 내어주도록 한다 (관리약사는 연구에 관련된 평가에 일체 관여하지 않을 예정이다).

연구에 참여한 피험자는 부여 받은 무작위 배정번호에 따라 미리 생성된 무작위 배정표대로, 연구에 참여하는 순차적으로 각 군에 배정되며, 각 군의 피험자 수 비는 1:1의 비율로 동일하게 한다.

**6. 6. 통계 분석 원칙 및 방법**

□ 대조군과 실험군, 두 군간에 primary endpoint인 장 정결도를 비교함에 있어, 장 정결도는 부적절(SNUH scale 1,2) 과 적절(3,4,5) 로 이분하여 분석한다. 각 군에서 부적절한 장정결(SNUH scale 1,2)의 비율을 를 이용하여 비교한다. 장 정결도는 눈가림된 expert reviewer에 의해 서울대학교병원대장정결도 등급에 따라 대장내시경 image를 평가하도록 한다. 두 군간 적절하게 이루어진 장 정결도를 비교하기 위해 Intent to treat analysis 및 Per protocol analysis를 시행한다.

□ 대조군과 실험군 간에 삽입 시간, 회수 시간을 Student’s t-test 를 이용해 비교 분석한다. 두 군간에 선종 발견율, 맹장 도달율 및 부적절한 장정결로 인해 재검을 권유 받은 환자의 비율은 chi-square test 를 이용해 비교한다.

□ 이전 대장내시경 검사의 시행 유무를 조사하여 양군간에 유의한 차이가 있는지 여부를 확인한다. 복용법 수행의 용이성은 용이, 어려움, 매우어려움의 3단계로 구분하여 조사하고, 이전 검사를 시행한 적이 있을 경우 이전 검사와 비교하였을 때 더 쉬움, 비슷함, 더 어려웠음의 3단계로 구분하여 조사한다. 양 군에서 각 비율을 비교하여 유의한 차이가 있는지 확인한다. 하제복용법에 대한 순응도는 전량 복용 (우수), 75% 이상복용 (양호), 75% 이하 복용 (불량)의 3단계로 구분, 조사하여 양군에서 각각의 비율을 비교한다.

이전 검사와 비교하였을 때 전처치 방법의 선호도는 10-point VAS scale 을 이용하여 조사하고 그 평균score를 비교하여 유의한 차이가 있는지 평가한다.

□ 이상의 통계적 처리는 의학전문 통계 프로그램인 SPSS for Windows 17.0(SPSS Inc., Chicago, IL, USA)을 이용하고 p값이 0.05 미만인 경우를 통계적 유의성이 있다고 판정한다.

**7. 관찰 항목 및 관찰 검사 방법**

**7. 1. 연구 진행 일정표**

| 관찰항목 | 기간 |  |
| --- | --- | --- |
|  | 외래방문시 | 대장내시경검사일 |
| 피험자동의 | O |  |
| 선정/제외기준 확인 | O |  |
| 대장내시경 전처치법 교육 | O |  |
| 장정결도 평가 |  | O |
| 설문지 작성 |  | O |
| 증례기록지 작성 |  | O |

**7. 2. 관찰항목**

**7. 2. 1. 대장내시경 장정결도**

대장내시경 정결도는 서울대학교병원 대장 정결 정도 등급을 이용하여 환자가 어느 군에 배정되었는지 모르는 상태의 소화기내과 의사에 의해 대장내시경 시행 후 결과 기록지에 기록된다.

(1) 대장 정결 정도 등급 (서울대학교병원)

5 : completely clear

4 : clear liquid present

3 : liquid and solid stool present that can be aspirated

2 : liquid and solid stool present that cannot be totally aspirated

1 : solid stool preventing visualization

대장 정결 정도가 전장에서 1~2등급인 경우 또는 임상 의사의 판단하에 장정결 불량으로 인해 대장내시경 재시행이 필요한 경우를 부적절한 장정결로 정의한다.

(2) 장정결 불량으로 인해 재시행 필요 여부도 결과 기록지에 기록된다.

(3) 발견된 용종의 위치와 수, 크기에 대해서도 결과지에 기록된다

(4) 대장내시경 삽입 시간 및 회수 시간

대장내시경 삽입 시간은 시술 시작부터 충수돌기 입구가 보이는 맹장의 사진을 찍을 때까지의 시간으로 정의한다.

회수 시간은 맹장에서 대장내시경을 회수하기 시작하여 환자에게서 대장내시경을 뺄 때까지의 시간으로 정의한다.

삽입 시간 및 회수 시간은 보조 테크니션에 의해 결과 기록지에 기록된다.

**7. 2. 2. 대장내시경 시행력 및 설문조사**

연구에 참여하는 환자들은 대장내시경 시행 당일에 이전에 대장내시경을 시행 받은 적이 있는지, 이전에 시행 받은 적이 있다면 있다면 당시에 시행한 장정결방법은 어떤 것이 었으며, 당시 만족도는 어떠하였는지, 이번에 복용한 하제 복용법에 대한 순응도, 수행의 용이성 및 주관적인 부작용과 만족도는 어떠하였는지에 대하여 설문지를 작성한다.

**8. 평가기준, 평가 방법 및 해석 방법**

**8. 1. 평가 기준**

**8. 1. 1. 1차 평가 변수**

서울대학교병원 대장 정결 정도 등급에 따른 장정결 정도.

그 중에서도, ‘전장 (Cecum 에서 rectum에 이르기까지)의 장정결 정도’로 평가

**8. 1. 2. 2차 평가 변수**

(1) 서울대학교병원 대장 정결 정도 등급에 따른

Right / Transverse / Left segment 별 장정결 정도

(2) 이전 대장내시경 검사 시행력 : 예, 아니오

(3) 하제복용법 수행 용이성 :

이번 검사의 하제 복용방법 :

용이 (easy), 어려움 (difficult), 매우 어려움 (very difficult)

이전 검사에 비교하였을 때, 이번 검사의 하제 복용방법 :

더 쉬움, 비슷함, 더 어려웠음

(4) 하제복용법에 대한 순응도 :

전량 복용시 우수 (excellent)

75% 이상 복용시 양호 (good)

75% 이하 복용시 불량 (poor)

(5) 이전 대장내시경 검사 시행력이 있는 경우, 이전 검사 전처치와 이번 임상시험

전처치를 비교하였을 때, 선호도 : 10-point VAS scale

**5점.**

**둘다 비슷하다**

**10점. 이번 전처치가**

**훨씬 낫다**

**0점. 이전 전처치가**

**훨씬 낫다**

0 1 2 3 4 5 6 7 8 9 10

(6) 주관적인 부작용 유무 및 정도 : 3-point verbal scale

없었다 (none), 경도의 부작용 (some), 심한 부작용 (many)

(7) 주관적인 부작용의 종류 :

구역, 구토, 복통, 복부불편 및 팽만감, 어지러움, 불면증, 기타

(8) 삽입 시간, 회수 시간, 선종 발견율, 맹장 도달율

(9) 부적절한 장정결로 인해 재검을 권유 받은 환자의 비율

**9. 연구의 윤리성 확보를 위한 방안**

본 연구는 2008년 59차 세계 의사회(WMA) 서울총회에서 개정된 인간을 대상으로 하는 의학 연구에 있어서의 윤리 원칙인 헬싱키선언을 준수하며 환자의 개인 정보나 개별적인 치료 정보가 본 연구의 목적 이외에 사용되는 일은 허용되지 않는다.

임상시험에 들어가기 전에 피험자에게 시험내용 및 효과, 안전성에 대한 모든 사항을 설명한 후 자발적으로 본 연구에 참여하겠다는 동의서를 받은 경우에 연구에 참여하도록 한다. 임상시험의 승인을 얻거나 승인 받은 임상시험을 변경하여 실시하고자 하는 경우, 임상 단계별로 계획서 또는 변경 계획서에 대해 임상시험심사위원회의 승인을 받는다. 승인 이전에 피험자를 임상시험에 참여시킬 수 없다.

환자의 의무기록번호는 주관 연구자의 책임하에 별도의 파일로 보관하며 이를 코드화(암호화)하여 본 연구와 관련된 자료에서 신상 확인이 불가능하도록 관리한다. 피험자의 신원을 파악할 수 있는 기록은 비밀로 보장될 것이며, 임상시험의 결과가 출판될 경우에도 피험자의 신원을 비밀상태로 유지한다.

본 시험에 관련된 모니터 및 점검자는 임상 시험계획서와 GCP의 준수 여부를 확인하고 본 시험의 모니터링과 점검 및 진행사항 관리를 위한 목적으로 기록을 열람할 수 있다.

**10. 참고 문헌**

1. Burke CA, Church JM. Enhancing the quality of colonoscopy: the importance of bowel purgatives. Gastrointest Endosc 2007; 66: 565–73.

2. Froehlich F, Wietlisbach V, Gonvers JJ, et al. Impact of colonic cleansing on quality and diagnostic yield of colonoscopy: the European Panel of Appropriateness of Gastrointestinal Endoscopy European multicenter study. Gastrointest Endosc. 2005;61:378–384.

3. Adams WJ, Meagher AP, Lubowski DZ, King DW. Bisacodyl reduces the volume of PEG solution required for bowel preparation. Dis Colon Rectum 1994; 37: 229–33.

4. Sharma VK, Chockalingham SK, Ugheoke EA, Kapur A, Ling PH, Howden CW. Prospective, randomized, controlled comparison of the use of polyethylene glycol electrolyte lavage

solution in four-liter versus two-liter volumes and pretreatment with either magnesium citrate or bisacodyl for colonoscopy preparation. Gastrointest Endosc 1998; 47: 167–71.

5. Ell C, Fischbach W, Bronisch HJ, Dertinger S, Layer P, Rünzi M, Schneider T, Kachel G, Grüger J, Köllinger M, Nagell W, Goerg KJ, Wanitschke R, Gruss HJ. Randomized trial of low-volume PEG solution versus standard PEG + electrolytes for bowel cleansing before colonoscopy. Am J Gastroenterol 2008; 103: 883–93.

6. MJ Kang, SA Jung, JM Jung, HJ Song, SE Kim, HK Jung, KN Shim, K Yoo, IH Moon. A Prospective Trial Comparing 4 L-Polyethylene Glycol with 2 L-Polyethylene Glycol Plus Bisacodyl Tablets for Colon Preparation 대한소화기내시경학회지 2008;37:167-173

7. Cohen LB, Sanyal SM, Von Althann C, Bodian C, Whitson M, Bamji N, Miller KM, Mavronicolas W, Burd S, Freedman J, Aisenberg J. Clinical trial: 2-L polyethylene glycol-based lavage solutions for colonoscopy preparation – a randomized, single-blind study of two formulations. Aliment Pharmacol Ther. 2010 Sep;32(5):637-44.

8. Brady CE, 3rd, Dipalma JA, Beck DE. Effect of bisacodyl on gut lavage cleansing for colonoscopy. Ann Clin Res 1987;19:34-8.
