# Supplementary material for: Comparable Efficacy of a 1-L PEG and Ascorbic Acid Solution Administered with Bisacodyl versus a 2-L PEG and Ascorbic Acid Solution for Colonoscopy Preparation: A Prospective, Randomized and Investigator-Blinded Trial
Source: PLoS One. 2016 Sep 2;11(9):e0162051. doi: 10.1371/journal.pone.0162051 (PMC5010253; doi:10.1371/journal.pone.0162051)
Supplement: S2 File — (DOCX) [file pone.0162051.s003.docx]

# **Study protocols**

**Comparison between 2L Coolprep® and Combination of 1L Coolprep® and bisacodyl as bowel preparation for colonoscopy**

**Date: July 2012**

**Seoul National university Hospital**

**Department of internal medicine**

**Division of Gastroenterology**

**Prof. Jong Pil Im**

# **STUDY SUMMARY**

| Title of protocol | Comparable efficacy of 1-L Ascorbic Acid mixed PEG with Bisacodyl versus 2-L Ascorbic Acid mixed PEG as Colonoscopy Preparation: A Prospective, Randomized and Investigator-blinded Trial. |
| --- | --- |
| Objectives | Two litres of polyethylene glycol (PEG) solution with ascorbic acid (Asc) can provide efficacy similar to that of standard 4 L PEG solution for colonoscopy preparation. In addition, oral bisacodyl (Bis) has been shown to reduce the volume of PEG solution for bowel preparation with comparable efficacy. This study aimed to compare the efficacy, tolerability and safety of PEG solution mixed with Asc in 2 L versus the combination of Bis + 1 L PEG + Asc. |
| Research institute | Seoul National University Hospital  Seoul National University Boramae Hospital |
| Principal investigator | Prof. Ji Won Kim  Prof. Jong Pil Im |
| Study population | The patients who received outpatient colonoscopy exam at Seoul National University Hospital and Seoul National University Boramae Hospital |
| Study duration | Date of IRB approval ~ July 31 2013 |
| Study design | This is a prospective, randomized, multi-centre, single-blind, non-inferiority trial. Participants scheduled for colonoscopy will be included and randomized to receive either 2 L PEG + Asc only (group A) or 20 mg Bis + 1 L PEG + Asc (group B). The patients will be allocated into 2 groups according to a random-number table. Concealed allocation will be accomplished through independent personnel who will not be involved in the colonoscopy procedure. The quality of bowel preparation will be assessed using the Boston Bowel Preparation Scale. Data’s for tolerance, compliance and adverse events were also gathered. |
| Expected result | The efficacy of 2 L of PEG containing ascorbic acid (Asc) was as efficacious as the conventional 4 L PEG method with better compliance. Meanwhile, it is possibly to say that Bis could be effectively used for reducing the required volume of preparation methods. In the present study, the investigators will compare the efficacy and tolerability of a 2 L PEG solution containing Asc (PEG + Asc) and the combination of oral Bis 20mg with 1 L PEG + Asc for colonoscopy preparation. The present investigators expect that the preparation with more reduced volume by Bis addition could demonstrate not-inferior efficacy than 2 L of the PEG and Asc. |

**Table of Contents**

1. Title of Protocols ---------------------------------------------------------------------- 4

2. Research Institution --------------------------------------------------------------------- 4

3. Principal investigator and co-investigators ----------------------------------------- 4

4. Background and Objectives ------------------------------------------------------------ 6

5. Study Duration ----------------------------------------------------------------------- 7

6. Study Design

6.1 Study Design Summary------------------------------------------------------- 7

6.2 Study medications --------------------------------------------------------- 7

6.3 Inclusion and exclusion criteria ------------------------------------------- 9

6.4 Sample size justification ------------------------------------------------- 10

6.5 Randomization -------------------------------------------------------------- 12

6.6 Statistics---------------------------------------------------------------------- 12

7. Items of observations and methods

7.1 Study Schedule --------------------------------------------------------- 13

7.2 Items of observation ------------------------------------------------------- 14

8. Assessment and interpretation method-------------------------------------------- 15

9. Ethics Statement -------------------------------------------------------------------- 17

10. References ------------------------------------------------------------------------ 17

**1. Title of protocols**

**Title**: Comparison between 2L CoolprepⓇ and combination of 1L CoolprepⓇ and bisacodyl as bowel preparation for colonoscopy

**2. Research institution**

Institution: Seoul National University Boramae Hospital

Address: 20, Boramae-ro 5-gil, Dongjak-gu, Seoul, 07061, Rep. of KOREA

Institution: Seoul National University Hospital

Address: 101, Daehak-ro, Jongno-gu, Seoul 03080, Rep. of KOREA

**3. Principal investigator and co-investigators**

Principal Investigator: Professor, Ji Won Kim

Department of Internal medicine, Seoul National University Boramae Hospital

Address: 20, Boramae-ro 5-gil, Dongjak-gu, Seoul, 07061, Rep. of KOREA

+82-02-870-2251

Principal Investigator: Professor Jong-pil Im

Department of Internal medicine, Seoul National University Hospital

Address: 101, Daehak-ro, Jongno-gu, Seoul 03080, Rep. of KOREA

+82-02-740-8112

Sub-Investigator: Professor, Seong-Joon Koh

Department of Internal medicine, Seoul National University Boramae Hospital

Address: 20, Boramae-ro 5-gil, Dongjak-gu, Seoul, 07061, Rep. of KOREA

+82-02-870-2251

Sub-Investigator: Fellow, Ji Eun Kwon

Department of Internal medicine, Seoul National University Hospital

Address: 101, Daehak-ro, Jongno-gu, Seoul 03080, Rep. of KOREA

+82-02-740-8112

Sub-Investigator: Professor, Su Hwan Kim

Department of Internal medicine, Seoul National University Boramae Hospital

Address: 20, Boramae-ro 5-gil, Dongjak-gu, Seoul, 07061, Rep. of KOREA

+82-02-870-2251

**4. Background and Objectives**

A good-quality bowel preparation is critical for the efficacy of colonoscopy [1-2]. If the bowel cleansing is inadequate, polyps and lesions can be missed, the electrocautery risk is increased, the scope insertion is difficult, the examination takes longer, and the whole process may need to be repeated or rescheduled.

Conventional bowel preparation using 4 L of polyethylene glycol (PEG) solution is associated with poor patient compliance and tolerability because of the large volume and disagreeable taste. To increase patient satisfaction and compliance, studies were conducted using the combination of a reduced volume of PEG and oral medicine. The efficacy of 2 L of PEG containing ascorbic acid (Asc) or oral bisacodyl (Bis) was as efficacious as the conventional 4 L PEG method with better compliance [3-7]. Currently, 2 L of the PEG and Asc combination is commonly used as a bowel cleanser, and this preparation shows comparable efficacy and better tolerability compared with 4 L PEG formulations [5].

Oral Bis, a stimulant laxative, is an unabsorbable diphenylmethane derivative [8]. The efficacy of bowel cleansing with 15 mg of Bis plus 2 L of PEG was not inferior to 4 L of PEG and showed better patient compliance and lower adverse events.[3]. Moreover, combining 20 mg of Bis with 2 L PEG solution showed better efficacy, improved patient satisfaction, and shortened bowel preparation time compared to 4 L of PEG solution [4].

Based on these results, oral Bis with a low volume of PEG is expected to improve the bowel cleansing effect and tolerability in colonoscopy preparation. In the present study, we will compare the efficacy and patient acceptability of a 2 L PEG solution containing Asc (PEG + Asc) and the combination of oral Bis with 1 L PEG + Asc for colonoscopy preparation. In addition, we will investigate patient satisfaction and compare adverse events for each bowel preparation.

**5. Study Duration**

Date of IRB approval ~ July 31 2013

**6. Study Design**

**6. 1. Study Design Summary**

This is a prospective, randomized, multi-centre, single-blind, non-inferiority trial. Participants scheduled for outpatient colonoscopy will be included and randomized to receive either 2 L PEG + Asc only (group A) or 1 L PEG + Asc plus 20 mg Bis (group B). The quality of bowel preparation will be assessed via endoscopic images by experienced endoscopists using the Boston Bowel Preparation Scale. The preparations will be also rated by the participants for tolerance, compliance, and adverse events.

**6. 2. Study medications**

Study medication for the present trial is 2L CoolprepⓇ (TaeJoon Pharmaceuticals, Seoul, Korea). CoolprepⓇ is mixture of PEG 3350, Anhydrous Sodium Sulfate, Sodium Chloride, Potassium Chloride, Ascorbic acid, Sodium L-Ascorbate, 2L CoolprepⓇ (TaeJoon Pharmaceuticals, Seoul, Korea). Related risk or adverse effect is fatigue, abdominal pain, bloating, anal irritation, nausea, vomiting, dizziness, and headache. For 1L of CoolprepⓇ contains chemical components as below.

1L of CoolprepⓇ: Sodium Chloride 2.691g, Potassium Chloride 1.015g, Anhydrous Sodium Sulfate 7.5g, PEG 3350 100.0g, Ascorbic acid 4.7g, Sodium L-Ascorbate 5.9g

Oral bisacodyl agent for the present trial is Dulcolax-SⓇ (Boehringer Ingelheim ,Seoul, Korea). Related risk or adverse event by Dulcolax-S is dehydration or electrolyte imbalance with just <1% risk. And mild abdominal pain, nausea, vomiting could be happened. For 1 table of Dulcorax-S contains Bisacodyl 5mg, docusate sodium 16.75mg

Drug interaction between Coolprep and Dulcolax-S was not ever reported. And the present investigator also confirmed by medical literature search including Launch Lexi-Interact™ Drug Interactions Program.

Meal before the day of colonoscopy is limited to semi-solid diet.

□ Medication for Control group (2 L of PEG + Asc)

Patients allocated to the 2 L of PEG + Asc group will take 250 ml of PEG + Asc at 15 min intervals to complete 1 L of the PEG + Asc solution at 8:00 PM of the day prior to the procedure and will be administered 1 L of the PEG + Asc solution in the same manner at 6:00 AM of the day of the procedure.

□ Medication for study group (20 mg Bis and 1 L of PEG + Asc group)

Patients in the 20 mg Bis and 1 L of PEG + Asc group will consume 20 mg of Bis with 500 ml of water at 8:00 PM of the day prior to the colonoscopy and will take 1 L of the PEG + Asc solution at 6:00 AM on the day of the colonoscopy in the same way as described above. All of the subjects will ingest 500 mL of water for every 1 L of PEG + Asc solution. The patients will be instructed to complete all of the administrations at least 3 hours before colonoscopy

**6. 3. Inclusion and exclusion criteria**

□ Inclusion Criteria

(1) Patients who agree to participate by the patient’s own will

(2) At least 20 years of age

(3) Patients who is scheduled for outpatient colonoscopy

□ Exclusion Criteria

(1) Prior abdomen-pelvic surgery

(2) Inflammatory bowel disease

(3) Ileus, suspected bowel obstruction or toxic megacolon

(4) Prior abdomen-pelvic surgery

(5) Severe cardiac, pulmonary, renal, hepatic or haematologic diseases

(6) Dementia or other cognitive disorders

(7) Patients with long-term use of tranquilizer, anti-spasmodics, prokinetics, laxatives or antidiarrhoeal agents

(8) Hypersensitivity to PEG solution or Bisacodyl

(9) Pregnant or breastfeeding woman

(10) Patient do not agree informed consent

□ Withdrawal or drop-out as an outcome measure

(1) If a violation of inclusion/exclusion criteria has occurred

(2) If a serious adverse events has occurred, or if the patient wanted to discontinue the trial because of adverse events

(3) If the patient decides to withdraw from this trial

(4) If the patient does not appear for their scheduled outpatient endoscopic procedure.

(5) If the patient had difficulties to take bowel preparation solutions and medicines

(6) If the patient took any drugs which can influence the trial results

(7) If a trial investigator found any safety issue, or any other reasons to stop the tiral

**6. 4. Sample size justification**

This is a prospective, randomized, multi-centre, single-blind, non-inferiority trial to compare the efficacy of “1 L PEG + Asc (CoolprepⓇ) + Bisacodyl” and “2 L PEG + Asc (CoolprepⓇ )”(one-sided test). Subjects will be randomly assigned in a 1:1 ratio at each participating site, and a non-inferiority margin of 15% will be used.

According to a United Kingdom study, successful gut cleansing was achieved in 136 of 153 (88.9%) cases of the 2L PEG + Asc group and 147 of 155 (94.8%) cases of the 4 L group [5], and by the USA study, successful gut cleansing rate was achieved 92% in 2L PEG + Asc group and 89% in 2L PEG + Bisacodyl group [7]. Moreover, combining 20 mg of Bis with 2 L PEG solution showed better efficacy, improved patient satisfaction, and shortened bowel preparation time (successful bowel preparation rate of 79.9%) compared to 4 L of PEG solution (successful bowel preparation rate: 96.6%), in a Korean study [6]

The amount and ingredients of CoolprepⓇ was equal to 2L PEG plus Ascorbic acid in previous studies, and the successful bowel preparation rate was 80% in Korean study despite 88%- 89% in worldwide studies. Based upon the previous studies, P_t_ was expected to be 85%, and a non-inferiority margin of 15% was used. Sample size calculation of this non-inferiority study was based on assumption of α=0.025 and β=0.8. (P_t_= 0.85, ε =0.15, α=0.025, β=0.8)

P_t_ : expected success rate of study group, P_c_ : expected success rate of control group, λ : assigned ratio between two groups (study group : control group=1: λ), ε : the difference in success rates,

This sample size was calculated using the following equation.


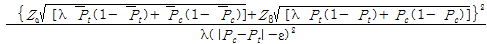


If, P_t_ =P_c_ =P, the equation is as follows.


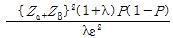


And, using the values described as above, the result is as fallows.
$\frac{(1.96 + 0.84)^{2} x (1+1) x 0.85 x (1-0.85)}{1 x ( 0.15 )^{2}}$ ≒ 88.853

As the result, we need to enroll 89 patients in each group. Considering a drop-out rate of 10%, the total sample size required was 196 (98 patients in each condition).

**6. 5. Randomization**

Calculated sample size was 196 in this study (98 patients in each group). A random-number table will be made by Medical Research collaborating center (MRCC). Using the random number table, the independent pharmacist will give the bowel preparation solution and medicines (The pharmacist will not be involved in the colonoscopy procedure or out-patient department work).

The enrolled subjects will be randomly assigned as a random number table in consecutive order, and the assigned ratio between two groups will be 1:1.

**6. 6. Statistical Consideration**

□ Primary end point : the rate of successful bowel preparation rate

According to the bowel preparation scale, BBPS scale (Boston Bowel Preparation Scale), the bowel preparation state will be assessed as below

0 = Unprepared colon segment with mucosa not seen due to solid stool that cannot be cleared.

1 = Portion of mucosa of the colon segment seen, but other areas of the colon segment not well seen due to staining, residual stool and/or opaque liquid.

2 = Minor amount of residual staining, small fragments of stool and/or opaque liquid, but mucosa of colon segment seen well.

3 = Entire mucosa of colon segment seen well with no residual staining, small fragments of stool or opaque liquid.

The successful bowel preparation rate will be assessed by blinded experts by review of all of the colonoscopic images. Intent to treat analysis and Per protocol analysis will be used.

□ Insertion time and withdrawl time will be assessed using Student’s t-test. Adenoma detection rate, cecal intubation rate, and failed colonoscopy rate caused by poor bowel preparation will be assessed by chi-square test.

□ If the patient experienced previous colonoscopy, the previous bowel preparation method will be assessed using questionnaire. The participants will be also asked to complete a questionnaire regarding the compliance, tolerability, adverse events of the bowel cleansing procedure, and the amount of preparation they completed (100%, 75~99%, 75% or less). The preference of the bowel preparation methods will be assessed using 10-point VAS scale.

□ All analyses were performed using the SPSS statistics program (version 17.0; SPSS Inc., Chicago, IL, USA). A P value less than 0.05 was considered statistically significant.

**7. Items of observations and methods**

**7. 1. Study Schedule**

| Items of observation | The day of |  |
| --- | --- | --- |
|  | OPD visit | Colonoscopy |
| Obtaining informed consent | O |  |
| Enrollment according to the  inclusion/exclusion criteria | O |  |
| Instructions for bowel preparation  as study protocol | O |  |
| Assessment of bowel preparation state |  | O |
| Questionnaire |  | O |
| Case report form |  | O |

**7. 2. Elements of measurement**

**7. 2. 1. Bowel preparation**

All of the involved endoscopists will be unaware of the patient’s bowel preparation method and take pictures of each bowel segment while the scope insertion before bowel content removal. And two experienced endoscopists, blinded to the preparation method, will review all of the colonoscopic images and bowel cleansing status is evaluated using BBPS scale (Boston Bowel Preparation Scale)

(1) BBPS scale (Boston Bowel Preparation Scale), the bowel preparation state will be assessed as below

0 = Unprepared colon segment with mucosa not seen due to solid stool that cannot be cleared.

1 = Portion of mucosa of the colon segment seen, but other areas of the colon segment not well seen due to staining, residual stool and/or opaque liquid.

2 = Minor amount of residual staining, small fragments of stool and/or opaque liquid, but mucosa of colon segment seen well.

3 = Entire mucosa of colon segment seen well with no residual staining, small fragments of stool or opaque liquid.

-defined the achievement adequacy if the subject has BBPS scores of 2 or 3 for all colon segments and inadequate if the subject has BBPS scores of 0 or 1 in any colon segment

(2) Whether repeat colonoscopy was recommended, the endoscopist will record the recommendation in result form.

(3) If the polyps detect, the location and size will be recorded.

(4) Insertion time and withdrawl time

Insertion time is defined as the time from insertion into the rectum to the time when the colonoscope tip passed to a point proximal to the ileocecal valve so that the base of cecum was visible.

Withdrawal time is defined as the time taken for withdrawing the colonoscope tip from the base of cecum to across the anus. Insertion and withdrawal times were recorded immediately after finishing the examination by the assistant.

**7. 2. 2. Questionnaire**

On the day of colonoscopy, participants will be asked to complete a questionnaire regarding their prior colonoscopy experience and the compliance, tolerability, and adverse events of the bowel cleansing procedure. The patients who experienced prior colonoscopy will be also asked about their preference to bowel preparation method between the prior regimen and the bowel preparation method in the present study.

**8. Assessment and interpretation method**

**8. 1. Endpoints**

**8. 1. 1. Primary end point**

Bowel preparation status, using BBPS scale

Achievement of adequacy and the overall colon cleansing score ≥ 6

**8. 1. 2. Secondary end points**

(1) Bowel preparation status, using BBPS scale

Bowel preparation status of right / transverse / left colon

(2) Experience of colonoscopy: Yes, No

(3) Patient tolerability :

The bowel preparation method difficulty:

easy, difficult, very difficult

than previous bowel preparation regimen (previous colonoscopy +) :

easier, no difference, more difficult

(4) the amount of preparation they completed:

100% (excellent)

75% ~99% (good)

75% or less (poor)

(5) Preference to bowel preparation method between the prior regimen and the trial regimen : 10-point VAS scale

**10 : the trial regimen is much better.**

**0 : the prior regimen is much better**

**5 :**

**No difference**

0 1 2 3 4 5 6 7 8 9 10

(6) The presence of distressing symptoms : 3-point verbal scale

none, some, many

(7) Distressing symptomes :

nausea, vomiting, abdominal pain, abdominal discomfort, dizziness, insomnia, etc

(8) Insertion and withdrawl times, adenoma detection rate, cecal intubation rate

(9) Proportion of repeat colonoscopy recommend patient (due to poor bowel preparation)

**9. Ethics Statement**

This study follows the Declaration of Helsinki on medical research protocols and ethics, (the 59^th^ World Medical Association, 2008) and the patient’s privacy and personal information will be protected.

Participation by individuals capable of giving informed consent as subjects in medical research must be voluntary. Before the enrollment, each potential subject must be adequately informed of the aims, methods, expected results and safety. For medical research using changed protocol, new protocol need to be approved by the institutional review board (IRB). Before the approval of IRB, the trial cannot start of recruitment.

Patient’s identification data will be will be stored safely at the principal investigator’s independent storage and kept separate from the data set used for analysis. All participants’ private informations will be protected before and after the publication.

As the study progresses, adherence to instrument and intervention manuals will be continuously monitored and training refreshed by the trial staff responsible for data collection and application of the intervention.

**10. References**

1. Burke CA, Church JM. Enhancing the quality of colonoscopy: the importance of bowel purgatives. Gastrointest Endosc 2007; 66: 565–73.

2. Froehlich F, Wietlisbach V, Gonvers JJ, et al. Impact of colonic cleansing on quality and diagnostic yield of colonoscopy: the European Panel of Appropriateness of Gastrointestinal Endoscopy European multicenter study. Gastrointest Endosc. 2005;61:378–384.

3. Adams WJ, Meagher AP, Lubowski DZ, King DW. Bisacodyl reduces the volume of PEG solution required for bowel preparation. Dis Colon Rectum 1994; 37: 229–33.

4. Sharma VK, Chockalingham SK, Ugheoke EA, Kapur A, Ling PH, Howden CW. Prospective, randomized, controlled comparison of the use of polyethylene glycol electrolyte lavage

solution in four-liter versus two-liter volumes and pretreatment with either magnesium citrate or bisacodyl for colonoscopy preparation. Gastrointest Endosc 1998; 47: 167–71.

5. Ell C, Fischbach W, Bronisch HJ, Dertinger S, Layer P, Rünzi M, Schneider T, Kachel G, Grüger J, Köllinger M, Nagell W, Goerg KJ, Wanitschke R, Gruss HJ. Randomized trial of low-volume PEG solution versus standard PEG + electrolytes for bowel cleansing before colonoscopy. Am J Gastroenterol 2008; 103: 883–93.

6. MJ Kang, SA Jung, JM Jung, HJ Song, SE Kim, HK Jung, KN Shim, K Yoo, IH Moon. A Prospective Trial Comparing 4 L-Polyethylene Glycol with 2 L-Polyethylene Glycol Plus Bisacodyl Tablets for Colon Preparation Korean J Gastrointest Endosc. 2008;37:167-173

7. Cohen LB, Sanyal SM, Von Althann C, Bodian C, Whitson M, Bamji N, Miller KM, Mavronicolas W, Burd S, Freedman J, Aisenberg J. Clinical trial: 2-L polyethylene glycol-based lavage solutions for colonoscopy preparation – a randomized, single-blind study of two formulations. Aliment Pharmacol Ther. 2010 Sep;32(5):637-44.

8. Brady CE, 3rd, Dipalma JA, Beck DE. Effect of bisacodyl on gut lavage cleansing for colonoscopy. Ann Clin Res 1987;19:34-8.
